# Durability of Resin Bonding to Dental 3Y-TZP Zirconia Using Different Adhesive Systems

**DOI:** 10.3390/ma17020424

**Published:** 2024-01-15

**Authors:** Christine Yazigi, Shila Alawi, Sebastian Wille, Frank Lehmann, Matthias Kern

**Affiliations:** Department of Prosthodontics, Propaedeutics and Dental Materials, School of Dentistry, Christian-Albrechts University at Kiel, 24105 Kiel, Germany; shila.alawi@hotmail.de (S.A.); swille@proth.uni-kiel.de (S.W.); flehmann@proth.uni-kiel.de (F.L.); mkern@proth.uni-kiel.de (M.K.)

**Keywords:** adhesive bonding, luting resins, MDP, primer, zirconia ceramic, self-adhesive luting resin, conventional luting resin

## Abstract

This laboratory study was conducted to evaluate and compare the resin bond strength of different adhesive resin systems in different combinations and the durability of their bonds with zirconia ceramic. Materials and methods: One hundred and twenty-eight specimens were milled from 3Y-TZP zirconia ceramic. The bonding surfaces of all disks were wet polished, steam cleaned, airborne-particle abraded and ultrasonically cleaned in 99% isopropanol. The specimens were randomly divided into four main groups according to the applied resin system; two conventional and two self-adhesive systems were used. Each group was further subdivided into two subgroups; the first was conditioned with the specified primer for conventional luting resins or not conditioned for the self-adhesive systems, whereas the second subgroup of each was conditioned with the same phosphate monomer-containing primer (Alloy Primer). The zirconia specimens were adhesively bonded, using the allocated luting resin, to plexiglass tubes filled with self-curing composite resin (Clearfil FII). Half of the specimens of each subgroup were stored in distilled water at 37 °C for 3 days, whereas the other half were subjected to artificial aging, 150 days of storage and additional thermal cycling. Thereafter, all specimens were subjected to TBS testing using a universal testing machine. Statistical analysis was conducted using two-way ANOVA followed by separate one-way ANOVAs. The Games–Howell post-hoc test was applied for pairwise comparisons. Results: All specimens survived storage with thermal cycling. The mean TBS values ranged from a minimum of 43.4 ± 5.0 MPa to a maximum of 66.4 ± 3.5 after 3 days and from a minimum of 13.6 ± 2.5 MPa to a maximum of 50.1 ± 9.4 MPa after 150 days. Conclusions: Artificial aging had a significantly negative effect on all test groups. The chosen adhesive-resin system had a significant effect on the resulting TBS values. The highest TBS values were achieved for the self-adhesive luting resin G-Cem One but were statistically comparable to the results obtained for the dual-cure luting resin G-Cem LinkForce.

## 1. Introduction

Advancements in computerized dentistry and the development of high-strength all-ceramic restorative materials in addition to reliable adhesive bonding systems contributed to the rise of the minimally invasive trend in the field of dentistry [1,2,3,4,5,6,7,8,9,10,11,12,13].

The concept of minimally invasive dentistry is based on defect-oriented conservative preparation that can save up to 40% of the intact tooth structure compared to the necessary preparation for full-coverage restorations [1]. The success of the adhesive-bonding technique facilitated the minimally invasive trend and led to a preference for the maximum conservation of sound tooth structure [1,2,3,4,5,6,7,8,9,10].

However, the survival and durability of these restorations rely, due to the lack of a retentive preparation form, on achieving strong and durable adhesive bonding between the tooth substrate and the luting resin and between the intaglio surface of the restoration and the luting resin. These bonds are influenced by many factors, such as the restorative material, the tooth substrate, the luting resin used, the bonding agents and the bonding technique [2,14,15].

3Y-TZP zirconia ceramic is widely used as a restorative material due to its functional and esthetic properties, which are especially beneficial for all-ceramic restorations in the posterior area [3,16,17,18,19]. Due to its superior mechanical properties, adhesive bonding is not obligatory for all-ceramic restorations if a retentive preparation is present [2]. However, adhesive bonding is favorable for zirconia minimally invasive restorations that lack a retentive preparation form [20].

Adhesive bonding is a well-established but sensitive and complex technique, particularly for non-retentive minimally invasive restorations [21,22,23,24,25,26]. The procedure necessitates choosing the best adhesive system for the particular clinical case, depending on the chosen restorative material and the underlying tooth substrate and strictly following the recommended protocol [21,27,28].

For adhesive bonding to zirconia surfaces, appropriate surface conditioning is required prior to the luting in order to establish stable and durable bonding with luting resins. Following the alumina airborne-particle abrasion, a primer containing a phosphate monomer, methylacryloyloxydecyl-dihydrogenphosphate (MDP), is necessary for activating the chemical interactions of the hydroxyl groups in the zirconia surface with the ester group of the MDP monomer, leading to a strong and durable chemical bonding [29,30,31,32]. The establishment of the aforementioned surface-treatment protocol resulted in a distinctive improvement in adhesion to zirconia [33].

Owing to the sensitivity of the adhesive-bonding technique and its elaborate procedure, some practitioners are discouraged from implementing it in their daily practice. Moreover, even though the adhesive-bonding technique is well established and widespread, inaccurate and inappropriate applications are still practiced by dental practitioners [34]. This conclusion is supported by the published results of a survey conducted in Germany in 2019. This survey showed that only 62% of the surveyed dentists used proper adhesive techniques for bonding to oxide ceramics in comparison to 38% for bonding to silicate ceramics [34].

In order to address this problem and simplify the workflow of the sensitive adhesive-bonding technique, many manufacturers have resorted to developing universal primers that can be applied to both silicate and oxide ceramics [35]. Moreover, some manufacturers offer universal self-adhesive luting resins that contain MDP monomers and silane coupling agents that can be applied to all restorative surfaces, eliminating the prerequisite of a separate primer and providing a one-step bonding technique [21,36,37,38,39,40,41]. While it might be helpful to simplify the adhesive-bonding procedure, the effectiveness of such self-adhesive resins with completely different components and their possible chemical interactions mixed into one composition has not been thoroughly addressed [2,24].

Therefore, the aim of this study was to evaluate and compare the resin bond strength of different adhesive systems and their durability to zirconia ceramics. The first null hypothesis was that the application of different luting resin adhesive systems and the utilization of different conditioning protocols would not affect the bond strength between resin and zirconia ceramic. The second null hypothesis was that the durability of the bonds would not be affected by the artificial aging process.

## 2. Materials and Methods

### 2.1. Specimen Preparation

One hundred and twenty-eight disk-shaped cylindrical specimens (diameter: 8 mm, height: 3.4 mm) were milled from 3Y-TZP zirconia blocks (ICE Zirkonia, ZirkonZahn, Gais, Italy).

The bonding surfaces of all disks were wet polished with 600-grit abrasive silicon carbide paper. All specimens were first steam cleaned; then, the bonding surfaces of the specimens were color-marked and abraded at a 10 mm distance with 50 µm-sized Al_2_O_3_ airborne-particles at a pressure of 1 bar until the marked color was completely removed. Then, the specimens were ultrasonically cleaned in 99% isopropanol for 3 min and dried with oil- and water-free air spray.

The specimens were randomly divided into four groups, according to the allocated luting resin used (*n* = 32): Group V (Panavia V5; Kuraray Noritake, Hattersheim am Main, Germany), Group S (Panavia SA Cement Universal; Kuraray Noritake), Group L (G-Cem LinkForce; GC International, Luzern, Switzerland) and Group O (G-Cem One; GC International).

Each group was further subdivided into two subgroups (*n* = 16), according to the following conditioning protocol. For the dual-cure luting resins (Panavia V5 and G-Cem LinkForce), the first subgroup of each (V1 and L1) was conditioned with the system’s specific primer (Clearfil Ceramic Primer Plus and G-Multi Primer, respectively). The primers were applied to the adherent surfaces of the specimens with a micro-tip applicator brush for 10 s. Thereafter, the adherent surface was dried with a mild oil- and water-free air spray. The second subgroup of each (V2 and L2) was conditioned with a phosphate monomer-containing primer (Alloy Primer; Kuraray Noritake Dental Inc.), which was applied for 10 s to the adherent surface with an applicator brush and left to dry. For the self-adhesive luting resin groups (Panavia SA and G-Cem One), the first subgroup of each (S1 and O1) was not conditioned, whereas the second subgroup (S2 and O2) was conditioned with the phosphate monomer-containing primer (Alloy Primer), as mentioned above. The chemical compositions of the luting resins and the primers used in this study as well as the corresponding manufacturers are listed in Table 1.

**Figure 1:**
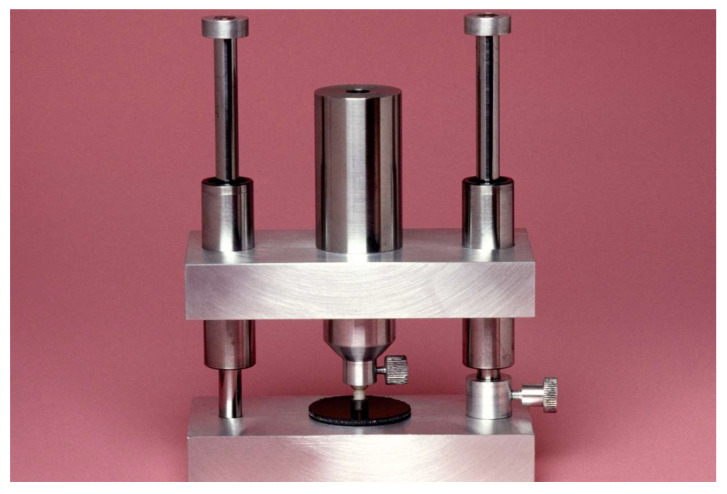
The alignment bonding apparatus consisting of parallel guides on both sides, a tube holder in the middle, a silicone pad and an added weight of 750 g.

One-hundred and twenty-eight plexiglass tubes (15.5 mm length and 3.2 mm inner diameter) were filled with self-curing composite (Clearfil FII; Kuraray Noritake), which was allowed seven minutes for auto-curing. The filled tubes were perpendicularly luted to the bonding surfaces using the allocated luting resin and a custom-made bonding apparatus with a weight of 750 g during the bonding procedure (Figure 1). The excess luting resin was gently removed by foam pellets, followed by polymerization for 20 s at 5 mm distance with an intensity of 1200 mW/cm^2^ (radii-cal; SDI Limited, Bayswater, Australia). The specimens were then light-cured for 90 s in a light polymerization unit (HiLite Power, Kulzer, Wehrheim, Germany) and then stored in an incubator at 37 °C.

### 2.2. Tensile Bond Strength Testing

**Figure 2:**
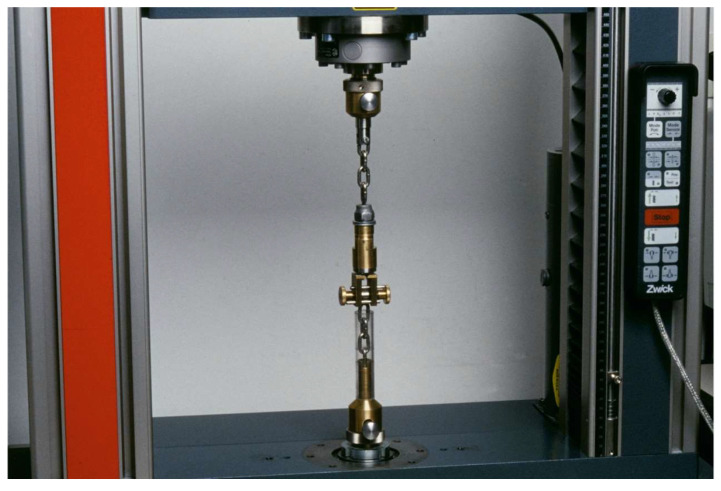
The universal testing machine and the construction of the TBS test.

**Figure 3:**
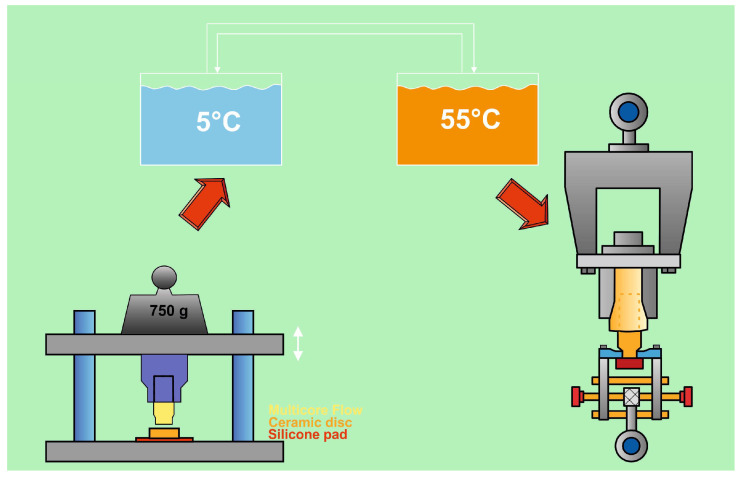
Workflow of the study.

Each subgroup was further randomly subdivided into two subgroups. The first subgroup was stored in water at 37 °C for three days. The second subgroup was subjected to artificial aging for 150 days with a total of 37,500 thermal cycles and temperature fluctuation (5 and 55 °C, 30 s dwell time). After the specified aging protocol was conducted, the bond strength of the luting resin was tested using a tensile bond strength test (TBS) with a universal testing machine (Zwick Z010; ZwickRoell Group, Ulm, Germany) [42] at a cross-head speed of 2 mm/min (Figure 2), where a moment-free axial application of the tensile force was enabled by means of a chain loop alignment. The TBS was calculated by dividing the force required to achieve debonding (F) by the bonding area ($A=\pi d^{2}/4$, d = 3.2 mm). The workflow of the study is illustrated in Figure 3.

### 2.3. Statistical Tests

The data were statistically analyzed (IBM SPSS Statistics, v20.0 for Windows; IBM Corp, Chicago, IL, USA). The data were tested for normal distribution using the Shapiro–Wilk test, which revealed that the data were normally distributed with the exception of group S1 (150 days). The homogeneity of the variances was tested with the Levene test, and proved to be significant (*p* = 0.004). Since the deviation from normal distribution implicated only one group, and considering that all groups had the same number of specimens, a two-way ANOVA was applied. A statistically significant interaction (*p* = 0.001) was revealed by the two-way ANOVA test between the variables (aging and treatment). Therefore, separate one-way ANOVAs were applied. The Games–Howell test was applied as a post hoc test for pairwise comparisons between groups regarding the effect of adhesive systems, as there was no homogeneity of variances given.

### 2.4. Failure Mode

After debonding, the ratio of adhesive and cohesive failure mode of each individual specimen was determined via optical microscopy. For this purpose, the bonding area without any visible residues on the ceramic surface was assigned to adhesive failure and bonding area with residues was assigned to cohesive failure. Additionally, a 10 nm thin gold layer was deposited via sputtering on a typical specimen of each group to obtain SEM images at a magnification of ×65 and an acceleration voltage of 10 kV (SEM, XL 30 CP; Philips, Eindhoven, The Netherlands).

## 3. Results

The mean values ± standard deviations of the TBS as well as the impact of aging on the mean TBS in % were calculated for each group, as shown in Table 2.

The mean TBS values after three days of water storage ranged from a minimum of 43.4 ± 5.0 MPa for group S1 to a maximum of 66.4 ± 3.5 MPa for group O1. All specimens survived storage and thermocycling for 150 days. The mean TBS values after 150 days ranged from a minimum of 13.6 ± 2.5 MPa for group V1 to a maximum of 50.1 ± 9.4 MPa for group O1. 

The artificial aging process with thermal cycling had a statistically significant negative effect (*p* < 0.05) on TBS values, as all test groups showed a significantly lower TBS after 150 days when compared to the initial TBS values obtained after three days.

The chosen resin system/conditioning protocol as a variable had a significant effect on the resulting TBS values both after 3 days and after 150 days. Group O1 showed the highest TBS values followed by group O2 after 3 and after 150 days. However, these values were statistically comparable to those obtained for groups L1 and L2 after 3 and 150 days. The most noticeable decrease in TBS values after artificial aging was for groups V1 and V2, which scored the lowest TBS values by far after 150 days of artificial aging.

**Figure 4:**
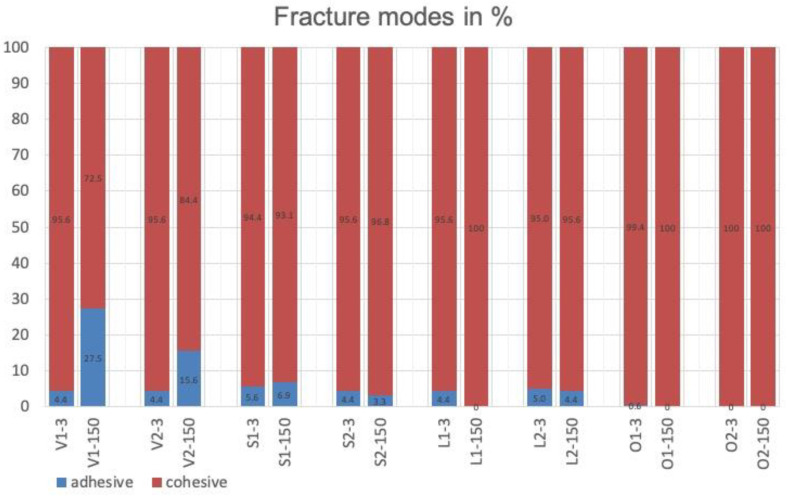
Percentages of areas assigned to failure mode for all test groups after TBS testing.

**Figure 5:**
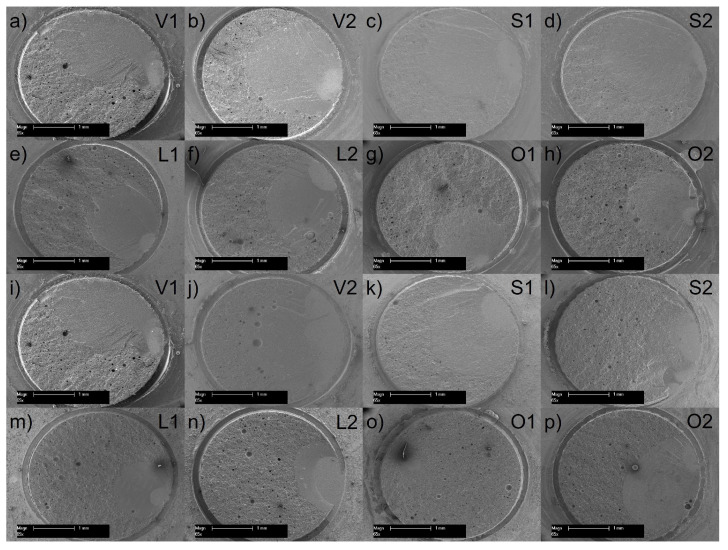
Representative scanning electron microscope images of the surface after debonding of a representative specimen of each group after: (**a**–**h**) 3 days water storage or (**i**–**p**) 150 days of water storage and thermocycling.

The failure-mode type is shown in Figure 4 for each group in percentages. The failure mode for groups L1 (150 days), O1 (150 days) and O2 (3 days and 150 days) was 100% cohesive. For all other groups the failure mode was also predominantly cohesive, which indicates debonded failures in the luting resin or in the resin composite. Representative SEM images are shown in Figure 5.

## 4. Discussion

The first null hypothesis was rejected, as the bond strength to zirconia ceramic was affected by the applied adhesive system and protocol. In this study, the highest TBS was obtained by group O for both subgroups (O1 and O2) and for both different allocated storage conditions. This indicates the favorable performance of the self-adhesive luting resin (GC-One), with subgroup O1 without any primer, as recommended by the manufacturer, performing slightly better than subgroup O2, which was conditioned using Alloy Primer. The results obtained by group L were statistically comparable to those of group O. Group V showed the lowest TBS values, statistically, after 150 days for both subgroups. 

It can be concluded that the chosen luting-resin system influenced the resulting bond strength for the same restorative material and that different resin-systems did not yield the same bonding values. This result is in accordance with the results of a study conducted by Blatz et al., where the bonding strength of different self-adhesive luting resins to zirconia was tested. However, in the aforementioned study, only self-adhesive luting resins were tested. Additionally, only half of the specimens were airborne-particle abraded. Moreover, a shear bond strength test was applied rather than the tensile bond strength applied in the current study. However, the resulting shear bond strength values varied between the different groups [43]. A study conducted by Passia et al. compared the TBS of three adhesive systems, one conventional luting resin combined with its universal primer and two universal bonding systems, in which one was tested once as a universal system without prior treatment and tested again when combined with a universal primer. The study concluded that the different bonding systems significantly affected the TBS, which is in accordance with the results of the current study [24].

This, however, contradicts the results of a study conducted by Samran et al., as the type of luting resin did not affect the resulting TBS for all tested systems with the exception of one [44]. The fact that only self-adhesive resins were tested in the aforementioned study could be a reason for this contradiction, although the performance of the two tested self-adhesive luting resins differed significantly in the current study.

In the current study, the highest tensile bond strength values were recorded for group O1 and O2 for both storage protocols, which indicates the statistically superior performance of the self-adhesive luting resin GC-ONE. However, when comparing the performance of the self-adhesive luting resins with the conventional luting resins that were tested in the current study, no conclusion could be drawn, as the results that were obtained for group O (self-adhesive) were comparable to those obtained for group L (conventional). Moreover, the lowest TBS values by far were recorded for group V (conventional) and were statistically comparable to those of group S (self-adhesive).

This is in accordance with the conclusions of the aforementioned study by Passia et al., in which two of the tested self-adhesive-resin systems yielded significantly higher TBS values after 150 days than those obtained for the conventional luting-resin group. However, the conventional luting resin performed significantly better when compared to the third tested self-adhesive luting resin [24].

A previous study [45] concentrating on the tensile bonding strength values for three restorative materials after contamination and after applying different cleaning methods showed a mean TBS of 24.4 MPa for the reference zirconia group that was primed with Ceramic Primer Plus and adhesively bonded with Panavia V5 after 150 days of artificial aging, which is higher than the mean TBS Value of 13.6 MPa obtained in the current study. The difference in TBS values, despite using the same protocol and the same aging and testing methods, may be attributable to the possible differences in the restorative materials’ compositions (ICE Zirkonia vs. Katana Zirconia STML). A recent study comparing the shear bonding strength of glass–ceramic-coated zirconia to alumina air-abraded zirconia after conditioning with G-Multi Primer and adhesive bonding with G-Cem LinkForce [46] showed SBS values of 32.0 MPa and of 26.3 MPa for the alumina air-abraded zirconia groups after 5,000 and 10,000 thermal cycles, respectively. This is lower than the 41.8 MPa achieved for the counterpart group in the current study after 150 days of artificial aging and 37,000 thermal cycles. The differences may be caused by the different testing methods (shear bond strength test vs. tensile bond strength), the different aging criterion and the possible differences in zirconia composition (GC Initial Zirconia HT Disk vs. Katana Zirconia STML). Another study focusing on the tensile bonding strength of different luting resins to zirconia with and without initial aging combined with different conditioning methods [47] showed that the group adhesively bonded with Panavia SA after alumina air-particle abrasion under a pressure of 1 bar and no initial aging, as in the current study, and had a median TBS value of 25.1 MPa after 150 days of artificial aging, which is comparable to the median value of 27.2 MPa obtained in the current study. These similarities are attributable to the application of the same conditioning protocols and the same aging and testing parameters. A laboratory study on the shear and tensile bonding strength of different luting resins to zirconia [48] concluded that only the group that was adhesively bonded with G-Cem Automix surpassed the threshold of a minimum tensile bond strength of 10 MPa after 90 days of storage in water. This excellent performance of G-Cem in the aforementioned study is in accordance with the conclusions of our study, despite the much higher TBS value recorded in our study.

According to previous studies, the minimum clinically acceptable bond strength range may be 10–13 MPa [49]. All adhesive systems and conditioning protocols tested in the study yielded bond strength values that were higher, although only slightly higher for some groups, than the minimum acceptable threshold values for bond strength.

Despite the relatively low bond strength of Panavia V5 after 150 days of storage in the current study, in a clinical study with non-retentive resin-bonded fixed dental prostheses with a mean observation time of nearly two years, no debondings occurred [50].

The second null hypothesis was rejected, as the TBS values for all test groups decreased significantly after artificial aging. This could be attributed to the deterioration and hydrolytic degradation of the bonding interface caused by water storage and thermal stress [44,51,52]. Water storage leads to the weakening of adhesive bonds caused by the diffusion of water molecules into the bonding interface [35,53]. Additionally, swelling stresses may arise from water absorption causing resin hydrothermal degradation [35,53]. Moreover, the variance in thermal coefficient expansion between the luting resin, the core composite resin and the zirconia caused by the application of thermal loading accelerates the degradation process [44,51,52].

This result is consistent with the findings of various previous studies, indicating the negative effect of artificial aging on the bonding strength of luting resin [24,30,43,44,45]. However, this conclusion is not in accordance with the findings of a study by Wolfart et al., in which only a minimal insignificant decrease in TBS was observed after thermocycling for 150 days for the group that was air-particle abraded and bonded with an MDP-containing composite resin [54]. This inconsistency regarding the effects of artificial aging despite using the same test methods and TBS test parameters may be explained by the different resin systems used. In addition, 50 µm alumina airborne-particle abrasion under a pressure of 2.5 bar was used in the previous study instead of the 1 bar applied in the current study.

In vitro laboratory studies attempt to mimic clinical conditions but cannot fully simulate intraoral conditions. The relatively low number of specimens in each group can be considered a limitation of the current study. Another limitation is the lack of saliva during the allocated storage period. The application of thermocycling with no mechanical fatigue during the artificial aging process might be considered another limitation to the study. The aforementioned limitations of the current study in addition to the possible different behavior of the tested adhesion materials and restorative materials in oral conditions compared to that in a laboratory study of an in vitro nature limit the generalizability of the findings of the current study and necessitate the use of controlled clinical trials to investigate the strength and durability of the achieved bond strength according to the adhesive system used as well as the conditioning method.

## 5. Conclusions

Within the limitations of this laboratory study, the following conclusions could be drawn:

1. The self-adhesive luting resin GC-ONE, as well as the conventional luting resin G-CEM LinkForce in combination with its specified primer G-Multi Primer, appeared most effective when bonding to zirconia and exhibited a stable performance after artificial aging.
2. The use of the phosphate monomer-containing primer (Alloy Primer) did not statistically enhance the performance of any of the tested bonding systems.
3. Artificial aging adversely affected the TBS values for all tested bonding systems.

## Figures and Tables

**Table 1:** Chemical composition of the luting resins and the primers tested in the study and the corresponding manufacturer.

Material	Composition	Manufacturer
**Ceramic Primer Plus**	3-Methacryloxypropyl trimethoxysilane, ethanol, 10-methacryloyloxydecyl dihydrogen phosphate (MDP)	Kuraray Noritake Dental Inc.
**Alloy Primer**	Acetone, 10-methacryloyloxydecyl dihydrogen phosphate (MDP), 6-(4-vinylbenzyl-n-propyl) amino-1,3,5-triazine-2,4-dithione (VBATDT)	Kuraray Noritake Dental Inc.
**G-MULTI PRIMER**	Ethanol, methacryloyloxydecyl dihydrogen phosphate (MDP), methacryloyloxydecyl dihydrogen thiophosphate (MDTP), γ-methacryloxypropyl trimethoxysilane (Silane), methacrylate monomer	GC International, Luzern, Switzerland
**G-CEM LinkForce**	Paste A: bis-GMA, urethanedimethacrylate, dimethacrylate, barium glass, initiator, pigments Paste B: bis-MEPP, urethanedimethacrylate, dimethacrylate, barium glass, initiator	GC International
**G-CEM** **One**	Paste A: fluoroaluminosilicate glass, UDMA, dimethacrylate, initiator, stabilizer, pigment, silicondioxide, MDP Paste B: SiO2, trimethoxysilane, UDMA, 2-hydroxy-1,3-dimethacryloxypropane, MDP, 6-tert-butyl-2,4-xylenol, 2,6-di-tert-butyl-p-cresol, EDTA disodium salt dehydrate, vanadyl acetylacetonate, TPO, ascorbic acid, camphorquinone, MgO	GC International
**Panavia SA**	Paste A: 10-methacryloyloxydecyl dihydrogen phosphate (MDP), bisphenol A diglycidylmethacrylate (Bis-GMA), triethyleneglycol dimethacrylate (TEGDMA), hydrophobic aromatic dimethacrylate, 2-hydroxymethacrylate (HEMA), silanated barium glass filler, silanated colloidal silica, dl-camphorquinone, peroxide, catalysts, pigments Paste B: hydrophobic aromatic dimethacrylate, silane coupling agent, silanated barium glass filler, aluminum oxide filler, surface-treated sodium fluoride (less than 1%), dl-camphorquinone, accelerators, pigments	Kuraray Noritake Dental Inc.
**Panavia V5**	Paste A: bisphenol A diglycidylmethacrylate (Bis-GMA), triethyleneglycol dimethacrylate (TEGDMA), hydrophobic aromatic dimethacrylate, hydrophilic aliphatic dimethacrylate, initiators, accelerators, silanated barium glass filler, silanated fluoroaluminosilicate glass filler, colloidal silica. Paste B: bisphenol A diglycidylmethacrylate (Bis-GMA), hydrophobic aromatic dimethacrylate, hydrophilic aliphatic dimethacrylate, silanated barium glass filler, silanated aluminum oxide filler, accelerators, dl-camphorquinone, pigments	Kuraray Noritake Dental Inc.

**Table 2:** Tensile bond strength with medians, means and standard deviations in MPa for all test groups; additionally, the relative impact on the mean TBS by aging is given.

Groups	3 Days		150 Days		
	Mean (SD)	Median	Mean (SD)	Median	Impact by Aging
V1	51.2 ± 5.7 ^a, BC^	50.1	13.6 ± 2.5 ^b, D^	13.7	−73%
V2	47.7 ± 6.3 ^a, C^	49.4	17.0 ± 3.6 ^b, CD^	16.4	−64%
S1	43.4 ± 5.0 ^a, C^	45.3	24.7 ± 7.7 ^b, BCD^	27.2	−43%
S2	45.8 ± 6.6 ^a, C^	44.6	28.3 ± 9.0 ^b, BC^	29.9	−38%
L1	60.2 ± 8.5 ^a, ABC^	61.1	41.8 ± 12.0 ^b, AB^	40.2	−31%
L2	59.5 ± 8.2 ^a, ABC^	61.7	36.1± 14.0 ^b, ABC^	35.3	−39%
O1	66.4 ± 3.5 ^a, A^	68.2	50.1 ± 9.4 ^b, A^	49.9	−25%
O2	62.0 ± 5.5 ^a, AB^	63.9	42.2 ± 5.0 ^b, A^	41.6	−32%

Different superscript uppercase letters indicate significant differences within each column. Different superscript lowercase letters indicate significant differences within each row.

## Data Availability

Data can be obtained by email request.
